# Editorial: Research on Emotion and Learning: Contributions from Latin America

**DOI:** 10.3389/fpsyg.2020.00585

**Published:** 2020-04-07

**Authors:** Camilo Hurtado-Parrado, Carlos Gantiva, Alexander Gómez-A, Lucas Cuenya, Leonardo Ortega, Javier L. Rico

**Affiliations:** ^1^Department of Psychology, Troy University, Troy, AL, United States; ^2^Faculty of Psychology, Fundación Universitaria Konrad Lorenz, Bogotá, Colombia; ^3^Department of Psychology, Universidad de los Andes, Bogotá, Colombia; ^4^Bowles Center for Alcohol Studies, University of North Carolina, Chapel Hill, NC, United States; ^5^Laboratorio de Psicología Experimental y Aplicada, Instituto de Investigaciones Médicas, IDIM – CONICET, Universidad de Buenos Aires, Buenos Aires, Argentina; ^6^Facultad de Psicología, Centro de Altos Estudios en Ciencias Humanas y de la Salud, Universidad Abierta Interamericana, Buenos Aires, Argentina; ^7^Facultad de Psicología, Instituto de Investigaciones, Universidad de Buenos Aires, Buenos Aires, Argentina

**Keywords:** learning, emotion, Latin America, research visibility, collaboration, academic productivity, research funding, Research and Development R&D

Contemporary research on emotions (“dispositions to action,” Lang, 1995), primarily focuses on emotion regulation (McRae and Gross, 2020), emotional intelligence (Gong and Jiao, 2019), and the relationship between emotion and social cognition (Stevens and Jovanovic, 2019). Technological advances have allowed researchers to identify the neurobiological processes underlying the emotions, in particular, functional magnetic resonance imaging (fMRI, Weber-Goericke and Muehlhan, 2019), electrophysiological techniques (Gantiva et al., 2019), and virtual reality (Wechsler et al., 2019). Notwithstanding these major progresses, there is awareness of the need to continue developing methodologies that further integrate behavioral measures and increase ecological validity. For example, using dynamic stimuli (e.g., videos) and not just photographs, as well as combining recording techniques that allow to understand neuronal activity underlying emotion over the course of time (e.g., EEG in conjunction with fMRI, Pavlova, 2017).

The study of emotions is currently one of the more developed fields in behavioral sciences, which is showing a high potential for integration across disciplines and research areas, for example trough computational models and creation of virtual reality environments (de Gelder, 2017). Basic and applied efforts to understand the interplay of emotion and learning are two of those fruitful areas of interaction (Hascher, 2010; Tyng et al., 2017; Wortha et al., 2019). Mounting evidence indicates that emotion modulates encoding and helps retrieval of information efficiently. Also, now there is evidence that emotion strongly affects attentional and executive processes (e.g., modulating selectivity of attention and motivating to action), which in turn are intimately linked to learning processes (Tyng et al., 2017).

Aligned with this global trend in research on emotion and learning, an important number of Latin American scientists have developed fruitful research agendas over the last decades. Their efforts have focused on basic and/or translational research with humans and other animal models, and on implementing diverse methodologies (Ardila et al., 2005; Annicchiarico et al., 2013; Robayo-Castro et al., 2016; Forero et al., 2020). Notwithstanding these important contributions, and efforts to develop regional and international networking systems (e.g., Red Latinoamericana de Ciencias del Comportamiento—https://redlacc.org; Sociedade Brasileira de Neurociências e Comportamento—http://www.sbnec.org.br/site/ and Asociación Latinoamericana de Neurociencias Aplicadas—http://www.neurolatam.org/index.php/en/), Latin American research on emotion and learning has followed the overall trend of other research fields throughout the region; namely, remaining partially hidden from the large scientific community of the world.

Although Latin America has increased its share of world scientific publications by nearly 2-fold during the last two decades, moving from ~2 to 4%, its output remains below the expected level based on its proportion of world population and Gross Domestic Product (GDP)—i.e., 5 to 6% (Van Noorden, 2014; Ciocca and Delgado, 2017). Moreover, recent analyses show that this growth has not been equally distributed across the region. While Brazil, Mexico, Argentina, Chile, and Colombia combined contribute to more than 90% of all scientific publications in the region, other neighboring countries remain behind the rest of the world (Huete-Perez, 2013). Ultimately, the scholarly impact of scientific research produced in the region remains below world average (e.g., citation rate remains below 1.0; Van Noorden, 2014). Two major interrelated factors that have contributed to this situation are that (a) most research groups tend to work in isolation or in local sporadic collaboration, and (b) results are often published in journals that are not indexed in key citation databases (e.g., SCOPUS or Web of Science; Van Noorden, 2014; Ciocca and Delgado, 2017).

In view of this situation, the present special issue entitled Research on Emotion and Learning: Contributions from Latin America aimed to engage researchers from the region to share empirical and conceptual work on emotion and learning in the journals Frontiers in Psychology and Frontiers in Education; two outlets that have high impact across different disciplines (emotion science, neuroscience, educational psychology, comparative psychology, health psychology, clinical psychology, and cognition). This effort was expected to result in strengthening and integrating our regional community of experts, enhancing global networking, and establishing new challenges and developments for future investigation.

The response to this initiative in the academic community was quite positive. Forty-six articles were submitted between November of 2017 and 2018, of which 33 were finally published. Most of these manuscripts were classified as *original research* (22); the remaining were distributed across *brief research reports* (5), *conceptual analyses* (2), *reviews* (2), *book reviews* (1) and *technology reports* (1).

An analysis of the published manuscripts keywords (Figure 1) indicates relationships to a wide range of topics. We observed differentiated emotion- and learning-related areas, covering basic and applied research with human and non-human species (e.g., rats, gerbils, birds, dogs) and expanding across various methodologies (e.g., psychophysiology, animal models, computer modeling). Worth noting is the fact that emotion-related topics were predominant, ranging from socially relevant areas, such as conflict and emotion regulation, to clinically and health-related areas, such as stress and psychopharmacology.

**Figure 1:**
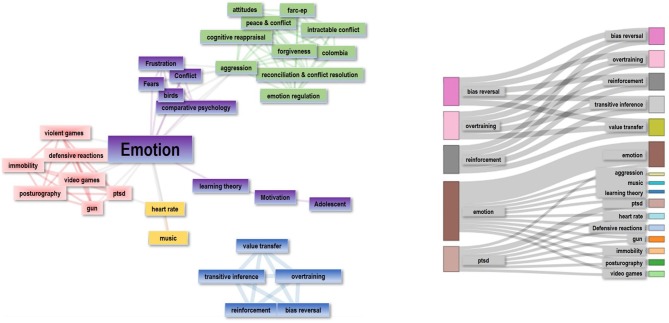
Keywords network of the articles published in the Research Topic entitled Research on Emotion and Learning: Contributions from Latin America. Relevance of the keyword is represented with larger nodes and wider connecting lines. Created with Bibliometrix (Aria and Cuccurullo, 2017).

Figure 2 shows the affiliation's country of published manuscripts and collaboration networks. Brazil, Argentina, Chile, Mexico, and Colombia were the only countries with contributions from Latin America. Unfortunately, this replicates the unequal pattern of scientific publications output previously identified in the region (Huete-Perez, 2013). In terms of collaboration, it practically only occurred between researchers with Latin American affiliations and institutions in other regions (e.g., United States, Canada, Spain, and Portugal). Collaboration between Latin American institutions was only observed in one instance (Colombia-Mexico). Both of these findings are aligned with previously reported patterns of collaboration (i.e., typically with non-Latin American institutions), which have been explained by the need of better visibility, research quality, impact factors, or international recognition (García et al., 2017).

**Figure 2:**
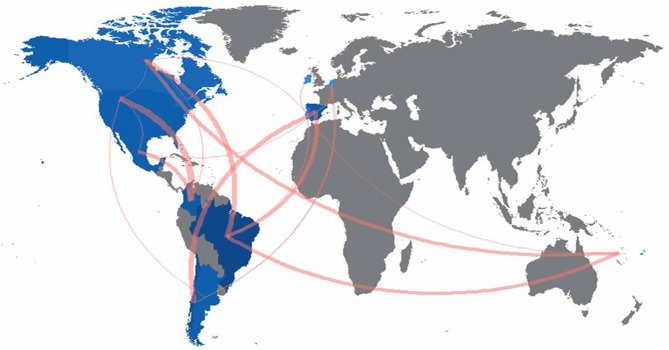
Collaboration network based on affiliation country of published manuscripts. Higher number of articles from a given country are represented with darker blue color. Wider connecting red lines indicate more collaboration. Created with Bibliometrix (Aria and Cuccurullo, 2017).

Regarding financial support, 85.29% of the published studies in this special issue reported funding by different sources, of which 65.51% corresponded to Latin-American Governmental agencies. In terms of authorship indicators, a total of 140 authors participated in the Research Topic. Only four papers were authored by a single person, and the rest (29) included two or more authors, with a mean of 4 authors per article (an additional indicator of collaboration).

Concerning the number of citations, as of January 2020 the average per manuscript published in the special issue was 0.7, which impressively approached citation's average worldwide (1.0) in a short period of time (Van Noorden, 2014). The top-three cited papers in the Research Topic came from authors of Brazil, Chile, Spain, Belgium, and the Netherlands. The first two were related to positive psychology and well-being, while the third one was related to defensive emotional reactions to violent video games.

Finally, an additional indicator of visibility is the total number of views that the articles included in the special issue have reached. In that regard, for instance, as of January 2020, Torres-Berrio et al.'s was positioned at the first quartile of visibility with up to 6,600 views in <2 years since the publication of that study. Ten other papers were positioned in quartile two, 13 papers in quartile three, and 10 papers in quartile 4 (Frontiers Loop®-https://loop.frontiersin.org/).

## Conclusions

When proposed, the aim of the present Research Topic was “to increase the visibility of Latin-American research on emotion and learning,” something that has been achieved in a relatively brief period of time. Researchers from Latin-American countries like Brazil, Chile, Colombia, Argentina, and Mexico, mainly (but not uniquely) interested in emotions and learning, shared on a large scale contemporary and relevant research across a wide range of basic, applied, and translational fields. They also showed a very representative net of collaborations between countries known for a strong tradition of research in North America, Europe, and Asia.

Another important aspect evidenced in the present special issue relates to the limited investment in science in Latin America. Funding information on the published manuscripts indicates that near half of the reported studies were supported with governmental resources. This somewhat resembles the low percentage of spending in Research and Development (R&D) in Latin-American countries (with the exception of Brazil, most countries in the region allocate on average <0.7% of the GDP—UNESCO Institute for Statistics—http://bit.ly/1USVQIP), as compared to that of developed countries in North America and Europe (beyond 2%—UNESCO Institute for Statistics—http://bit.ly/1USVQIP). This is a key point of interest for Latin-American researchers and their governmental agencies; namely, despite the low access to resources, researchers in the region are still capable of producing high-quality science. This is something that experts working on public policies need to “keep on the table” in order to continue advocating for increments in the resources that are greatly needed to support science in our countries.

Lastly, an aspect to highlight is the impact and visibility that most of the published articles are showing shortly after being released. This is aligned with previously reported growth in Latin-American science, as well as the recognition of its quality and applicability in other regions (Huete-Perez, 2013; Van Noorden, 2014; Ciocca and Delgado, 2017). Less than 1 year after the completion of the Research Topic, average citation per article is very close to reach worldwide standards (1.0 citation per article; Van Noorden, 2014).

## Author Contributions

All authors contributed to the conception and design of the project. CH-P and CG wrote the first draft of the manuscript. AG-A, LC, LO, and JR edited and added sections of the manuscript. All authors contributed to manuscript revision, read, and approved the submitted version.

### Conflict of Interest

The authors declare that the research was conducted in the absence of any commercial or financial relationships that could be construed as a potential conflict of interest.
